# Comparison of Rein Forces and Pressure Beneath the Noseband and Headpiece of a Snaffle Bridle and a Double Bridle

**DOI:** 10.3390/ani15071058

**Published:** 2025-04-05

**Authors:** Russell MacKechnie-Guire, Hilary Clayton, Jane Williams, David Marlin, Mark Fisher, Diana Fisher, Victoria Walker, Rachel C. Murray

**Affiliations:** 1 Equestrian Performance Research Centre, Hartpury University, Hartpury, Gloucester GL19 3BE, UK; jane.williams@hartpury.ac.uk (J.W.); victoria.walker@hartpury.ac.uk (V.W.); 2 Department of Large Animal Clinical Sciences, College of Veterinary Medicine, Michigan State University, 736 Wilson Road, East Lansing, MI 48824, USA; claytonh@msu.edu; 3 Animalweb Ltd., The Granary, Hermitage Court, Maidstone, Kent ME16 9NT, UK; dm@davidmarlin.co.uk; 4 Woolcroft Equine Services, May Lane, Wisbech IP30 0DQ, UK; woolcroft2002@yahoo.co.uk (M.F.); dianafisher007@yahoo.co.uk (D.F.); 5 Ibikus Ltd., Bury St Edmunds IP32 7AR, UK

**Keywords:** dressage, welfare, bridoon, curb, bridle pressure, rein tension, equestrian sport, bit

## Abstract

Information describing forces applied to the horse are needed to inform regulatory decisions regarding equine health and wellbeing. This study compares forces exerted beneath the noseband and headpiece of a snaffle bridle (SB) and a double bridle (DB). Horses were fitted with the same type of SB and DB. Forces were measured by pressure mats under the noseband (nasal/mandibular) and headpiece (occipital) of the bridle and by force sensors inserted bilaterally between the bit(s) and reins. The noseband was adjusted to 2 finger-equivalents using a tightness gauge. Data were recorded for eleven high-level dressage horses ridden in SB and DB in random order at collected walk, trot (sitting), and canter. The noseband pressures were similar between bridle types. Minimal, maximal, and mean occipital force and pressure were significantly higher for DB at walk, trot, and canter (all *p* ≤ 0.01), except minimal force for collected canter (*p* = 0.04). The rein tension for the bridoon bit alone and for the combined bridoon and curb bits was significantly lower than for the snaffle bit. Similar forces occur when ridden in SB and DB except that occipital force and pressure are higher due to the greater weight of the DB, and rein tension is lower for the DB.

## Simple Summary

In equestrian sports, pressure is applied to the horse via equipment and the rider’s aids, and neither should be a source of discomfort. This study measures and compares forces beneath a snaffle bridle (SB; single bit) and a heavier double bridle (DB; curb and bridoon). Eleven high-level dressage horses were fitted with an SB and DB in a random order. Pressure mats under the noseband (nasal, mandibular) and headpiece (occipital) measured compressive forces and pressure distribution. Sensors inserted between each rein and the bit(s) measured tensile forces. Few differences in noseband forces between bridles occurred. The greater weight of the DB applied higher minimal, maximal, and mean occipital forces. Rein tension did not differ between the snaffle bit of the SB and the summed forces of the bridoon and curb bits of the DB in walk/canter, but were lower for the DB when in collected trot. The force applied to the curb was less than the bridoon, and forces on each bit of the DB were less than for the SB. These findings indicate that in high-level horses, similar forces occur in an SB and DB except for occipital force and pressure, where the higher DB readings were due to greater weight.

## 1. Introduction

Dressage competition rules require the use of a bridle. A snaffle bridle (SB) is generally used at lower levels of training and may progress to the use of a double bridle (DB) as training progresses. Having the horse ‘on the bit’ is a key concept in the relationship between horse, bridle, and rider. Factors influencing this three-way relationship include the type of bridle and its adjustment [1,2,3,4], the rider’s skill set, the horse’s training level, and the gait and movement being performed. In contrast to the plethora of information describing saddle fit and effects on performance, relatively little has been published regarding the force and pressure exerted by the bridle and, more particularly, the double bridle.

Bridles are broadly divided into two types according to the number of bits. The SB has a single, non-leverage bit that may be unjointed, or single- or double-jointed. The DB has two bits: a bridoon and a curb. The bridoon is a non-leverage bit with one or two joints and acts similarly to a snaffle. The curb bit is an unjointed leverage bit that may have a central elevation in the mouthpiece for tongue relief. Due to the addition of the curb bit and its cheekpieces, a DB is heavier than an SB. The headpiece transfers the weight of the bridle to the horse’s head [5,6].

The bridle–horse interaction in dressage is under scrutiny [7,8], with the effect of noseband tightness receiving considerable attention [2,9,10,11,12,13,14,15,16,17,18]. Measurements using pressure mats have shown cyclic patterns of force and pressure on the horse’s face driven by the effects of gravity, inertia, and ground reaction forces on the noseband [3,19]. One study reported that horses showed peaks in noseband pressure during early stance when wearing a DB [3] but pressure peaks occurred closer to mid-stance when wearing an SB [19]. Mandibular pressure is higher and more variable than nasal pressure [19]. A Swedish noseband, described by other authors as “jaw clamping” [2], exerted only low-to-moderate pressure on the nasal bones and mandibular rami, with no difference in pressure values when compared to a cavesson or flash noseband in well-fitted bridles adjusted from 2.0 to 0.5 finger-equivalents tightness [19]. The noseband is not the only part of the bridle that exerts pressure on the horse’s face. The headpiece distributes the weight of the bridle across the occipital region. A maximal occipital force and pressure of 256 N and 46 kPa, respectively, have been reported beneath the headpiece in horses ridden in sitting trot, fitted with a DB [3]. However, there have been no direct comparisons between forces and pressures for SB and DB and no measurements reported for gaits other than trot.

Rein tension, in combination with other aids, influences the horse’s speed, direction, posture, and balance. During locomotion, the horse’s head and neck move in a gait-specific pattern and rider kinematics should accommodate and follow the movements of the horse. There are small changes in the distance from the rider’s hand to the bit that are the source of the characteristic pattern and frequency of rein tension in each gait [20,21]. In a study of horses ridden at trot, for example, movements of the rider’s trunk, shoulder, and elbow were synchronised such that the distance from the rider’s wrist to the horse’s bit changed by only 1.5 cm during the stride [22]. This is typically associated with rein tension oscillations of 20–40 N [23,24]. Rein tensions have been reported for horses wearing an SB in walk, trot, and canter when performing halts and transitions [25], and during a dressage training session [26,27]. However, little is known about rein tension values for a DB in high-level horses and riders.

The use of a DB is suggested to offer the rider a more refined level of communication with the horse by the use of two different bits in the mouth, which can act at different locations or in different directions to provide additional explanation to the horse about head position or movement. It has also been suggested that the DB may be used to apply greater forces to the horse. The horse should be familiar with and accept the action of the snaffle bit before introducing a DB [28] and the rider should have developed a high level of skill in order to use the bridoon and curb reins independently and correctly [29]. From a biomechanical standpoint, the presence of two bits with two sets of cheekpieces and a curb chain adds to the weight of the bridle. To the authors’ knowledge, neither the effects of the extra weight of a DB vs. an SB on facial force and pressure nor the effects of applying rein tension to the two bits of the DB have been investigated.

The objectives of the present study were to measure and compare force and pressure beneath the noseband and headpiece of the bridle and rein tension in high-level dressage horses and riders performing collected walk, collected trot (sitting), and collected canter wearing an SB compared with a DB. The hypotheses were first, that maximal total force and pressure on the occipital region would be higher when wearing a DB; second, that maximal nasal and mandibular force and pressure would not differ between the SB and DB; and third, that total rein tension would be higher with a DB than an SB.

## 2. Materials and Methods

An a *priori sample* size calculation (G*Power) was performed using mean and standard deviation for paired differences for maximal pressure data collected beneath the noseband in Murray et al. (2015) [3]. This estimated that for a sample size of 8 horses, the study’s power would be 0.90. Ethics were approved by Hartpury University’s Ethics Committee URN 2021-126. Written confirmation was provided by the Home Office, United Kingdom (February 2023), that the study did not require regulation under the Animals (Science Procedure) Act 1986 (ASPA). Informed, written consent was obtained from riders and owners prior to participation, and they were advised that they could withdraw from the study at any point.

### 2.1. Horses

Eleven high-level dressage horses competing at Prix St. Georges (PSG) and above with a mean (±standard deviation) height at the withers of 171 ± 1 cm, body mass of 545 ± 60 kg, and age 13 ± 4 years were recruited. The horses were housed at two locations, and all were part of an extensive equine sports science and medicine program, including regular therapy, dentistry, and veterinary assessments. As part of the inclusion criteria for the study, horses were required to have received a full dental check by a veterinarian or a qualified equine dental technician within three months prior to the study. As part of the horse’s training program, all horses were regularly ridden in an SB (~4 times/week) or DB (~3 times/week). On the day of the study, all horses underwent a clinical examination by a veterinarian (RM), where an absence of epaxial muscle tension was confirmed by digital palpation, along with an external examination of the horse’s mouth, lips, and commissures confirming the absence of oral lesions. After which, a gait evaluation in walk and trot was undertaken by a veterinary specialist prior to data collection to confirm that all horses included in the study had no lameness (all horses were graded 0 on a 0–10 grading scale).

### 2.2. Riders

Seven right-handed dressage riders (Prix St. Georges, Intermediate II, and Grand Prix) were recruited with a mean body mass of 68 ± 11 kg. Each horse was ridden by its usual rider.

### 2.3. Bridles and Bits

**Figure 1:**
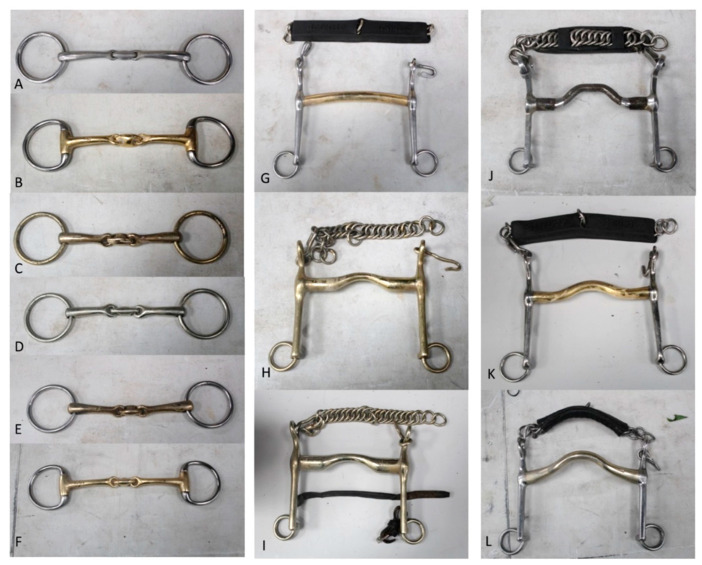
Types of mouth pieces used in the study. Snaffles (**A**–**C**), bridoons (**D**–**F**), and curbs (**G**–**L**). The type of curb chain, with or without a cover and/or lip strap, are included in (**G**–**L**).

Bridle fit was assessed by two Society of Master Saddlers-qualified bridle fitters. Horses wore their usual SB weighing 1.04 ± 0.20 kg and DB weighing 1.90 ± 0.20 kg, both of which were part of the horse’s general tack and equipment when being ridden. The bridles featured a headpiece shaped around the caudal margin of the ears and had attachments for the cheekpieces, noseband, and throat lash. All horses were ridden in a Swedish (crank) noseband, adjusted to a tightness of two finger-equivalents, determined by an International Society of Equitation Society (ISES) taper gauge [2] positioned over the midline of the nasal bone. The noseband type was the same for both bridles. For the SB, all horses regularly used a double-jointed snaffle mouthpiece (width: 12.0–15.2 cm). For the DB, each horse wore its usual bridoon (width: 13.0–15.2 cm) and curb (width: 12.0–14.6 cm). The lower-shank length of the curb was 10.1–13.9 cm with a simple curb chain (n = 7), a curb chain with rubber casing [3], or a leather guard [1] and/or lip strap (n = 1); all curb chains were fitted to the same level of laxity as used during training (Figure 1).

### 2.4. Pressure Mats

**Figure 2:**
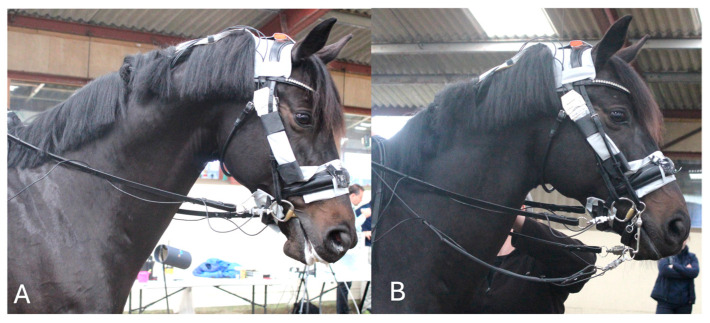
Position of the nasal, mandibular, and occipital pressure mats and rein tension devices fitted to the snaffle bridle (**A**) and double bridle (**B**).

Three small pressure mats were used (Pliance; Novel gmbh™, Germany): two noseband mats with sensor dimensions of 160 × 40 mm^2^ and an area of 64 cm^2^, with 64, 10 × 10 mm^2^ sensors/mat in a 16 × 4 configuration, and a resolution of 1 sensor/cm^2^; and a headpiece mat with sensor dimensions of 424 × 113 mm and an area of 479 cm^2^, with 240, 14.1 × 14.1 mm^2^ sensors/mat in a 30 × 8 configuration, and a resolution of 0.5 sensors/cm^2^. The noseband mats were positioned mid-dorsally over the nasal bones and mid-ventrally beneath the mandibular rami and the headpiece mat was placed mid-dorsally behind the ears (Figure 2).

The pressure mats were calibrated using a Trublu Calibration device (Pliance; Novel gmbh™, Germany), which loads each sensor over the entire pressure range, with the resultant adjustment curves stored for each sensor prior to the study within a range of 5–240 kPa, by shaping the bridle and pressure mats around a plastic model of a horse head, then initialising them to zero. The bridle and noseband shaped to match the model horse head were adjusted carefully to maintain the position of the pressure mats relative to the bridle. Cables from the pressure mats were braided into the horse’s mane and connected to a data logger attached to a saddle cloth. A 50 Hz video camera was hardwired to the laptop and synchronised with the three pressure mats. Data acquisition (pressure and video) was initiated and terminated within the Pliance system (Novel).

### 2.5. Rein Tension Sensors

Rein tensions were recorded using two pairs of miniature s-beam load cells with a range of 0 to 500 N (0–10 VDC output range; DBBSMM-50kg-002-000, Applied Measurements Ltd., Aldermaston, Berkshire, RG7 8PN, UK). Each load cell weighed 25 g and measured 35 × 24 × 12 mm. An M6 eye bolt (20 mm long, eye diameter 5 mm) was screwed into each end of the s-beam and locked with an M6 bolt and washer. One end of the load cell was connected directly to the bit ring with a 4 mm wide nylon cable fastener. The other end of the load cell was connected to the rein by a 32 mm diameter × 3 mm thick stainless-steel circular split ring. Each load cell was connected by a 2 m cable to a wireless strain gauge transmitter with battery power supply (T24-ACMI-SA, Applied Measurements Ltd., Aldermaston, Berkshire, RG7 8PN, UK). The cables were taped along the reins and the wireless strain gauge units were housed on the saddle pad in Velcro pouches.

Prior to the start of the study, each load cell was returned to the manufacturer for recalibration in a single direction, traceable to UKAS Standards. In addition, on the measurement days, the calibration and linearity of each load cell were verified before and after each treatment (horse and bridle) at 0 and 8 kg (80 N). The analogue wireless signals from the load cells were received by separate wireless receiver units with a range of 800 m and 0–10 VDC outputs per channel (T24-AO1i, Applied Measurements Ltd., Aldermaston, Berkshire, RG7 8PN, UK). The analogue outputs from the wireless receiver were digitised at 100 Hz by a 16-bit A/D convertor (USB-1608F-Plus, Measurement Systems Ltd., 16 Kingfisher Court, Newbury, Berkshire, RG14 5SJ, UK) and the data recorded using the DAQami software version 4.2.1f0 (Measurement Computing Corporation, Norton, MA 02766, USA). This configuration provided a resolution of <0.1 N. Recordings were exported as CSV files for further analysis in Microsoft Excel and Sigview 6.2.0.0 Pro (SignalLab e.K., Karl-Abt Straße 5, 75173 Pforzheim, Germany).

### 2.6. Study Protocol

After horses were fitted with the experimental equipment, a 10 min rider-prescribed warm-up was ridden that included walk, trot, and canter on the left and right reins. Six horses were ridden first in the SB and the remaining five horses were ridden first in the DB, once the data were collected, either the SB or DB were fitted and the riding protocol was repeated. For each bridle, data were collected while the horses were ridden in collected walk, collected trot (sitting), and collected canter along a 30.0 × 1.5 m track defined by small cones on the arena surface at 2 m intervals. Speed gates (Bower Timing Systems, Draper, UT, USA) were positioned at both ends of the track to measure the speed of the trial, and were transmitted wirelessly to a handheld unit. Entry to the experimental track was alternated between clockwise (right turn) and anti-clockwise (left turn) directions. The length of the track was sufficient to collect data for 12 strides at collected walk and 10 strides at collected trot or collected canter. Left and right trials that differed by ±0.2 m/s were discarded [30]; across all horses, 2 ± 1 trials were repeated. A veterinarian observed the behaviour of all horses at all times for indicators of pain or discomfort [31] or signs of lameness. No trials were terminated due to adverse behaviour or lameness. After completion of the trials, the horse was cooled down, all equipment was removed, and the horse was returned to its stable.

### 2.7. Data Processing

Pressure mats: For each trial, 12 consecutive strides of collected walk or 10 consecutive strides of collected trot or collected canter were analysed. Mean, maximal, and minimal force (N) and pressure (kPa) over all loaded sensors were determined on a stride-by-stride basis, exported to Signal View™, Mannheim, Germany, and filtered using a low-pass 10 Hz filter. Preliminary analysis indicated no significant differences in pressure variables when horses approached the runway on the left vs. the right rein (all *p* ≥ 0.05). Therefore, data for the two directions were pooled for further analysis.

Rein tension: No noise was apparent on any of the recorded signals and so no filtering was applied. Raw signals were imported into Excel and Macros used to organise data for further analysis within Excel or for export to Sigview. Within Excel, total instantaneous tension for the double bridle was determined by summing each timepoint (0.01 s) for the left and right bridoon or left and right curb. For the snaffle, total instantaneous tension was determined by summing the left and right snaffle tension. The complete recording for each rein was exported to Sigview where the mean, RMS, SD, minimal, and maximal values were determined for each 10 s recording. The Sigview peak detection routine was then used to calculate the number of peaks in each rein using the settings of RMS for peak threshold, minimum peak width = 0.1 s, and “detect multiple peaks within a single crossing” function set to “on”. The mean of the detected peaks was calculated and the number of peaks per second determined after division by the time from the horse passing between the start and end timing gates set 30 m apart. Thus, the distance travelled was always the same but the duration varied due to horse and gait. The % coefficient of variation for the whole signal was calculated as mean tension/SD tension × 100 and the % coefficient of variation for the peak signals was calculated as mean peak tension/SD peak tension × 100. Since this study measured forces on the horse’s head via the bridle, tension in the left and right reins was combined at each timepoint, so the rein tension values reported are the values summed over the two or four reins.

### 2.8. Statistics

Data were processed using SPSS Version 29. The Kolmogorov–Smirnov test indicated that the kinetic (noseband and headpiece forces and pressures), rein tension measurements, and kinematic (IMU) data distribution did not approximate a normal distribution. Therefore, a Wilcoxon signed-rank test was performed to investigate differences in the variables between the DB and SB. Median data for all outcome parameters with 25th and 75th percentiles is presented. Since this study included repeated measures, a Bonferroni adjustment was applied, resulting in a revised alpha of *p* ≤ 0.02.

## 3. Results

### 3.1. Collected Walk

#### 3.1.1. Bridle Forces

Table 1 compares median [25th and 75th] percentiles for the mean, maximal, and minimal forces on the nasal, mandibular, and occipital sites for the SB and DB at collected walk. There are significant differences between mean, maximal, and minimal occipital forces (*p* = 0.001, *p* = 0.010, *p* = 0.009, respectively).

**Figure 3:**
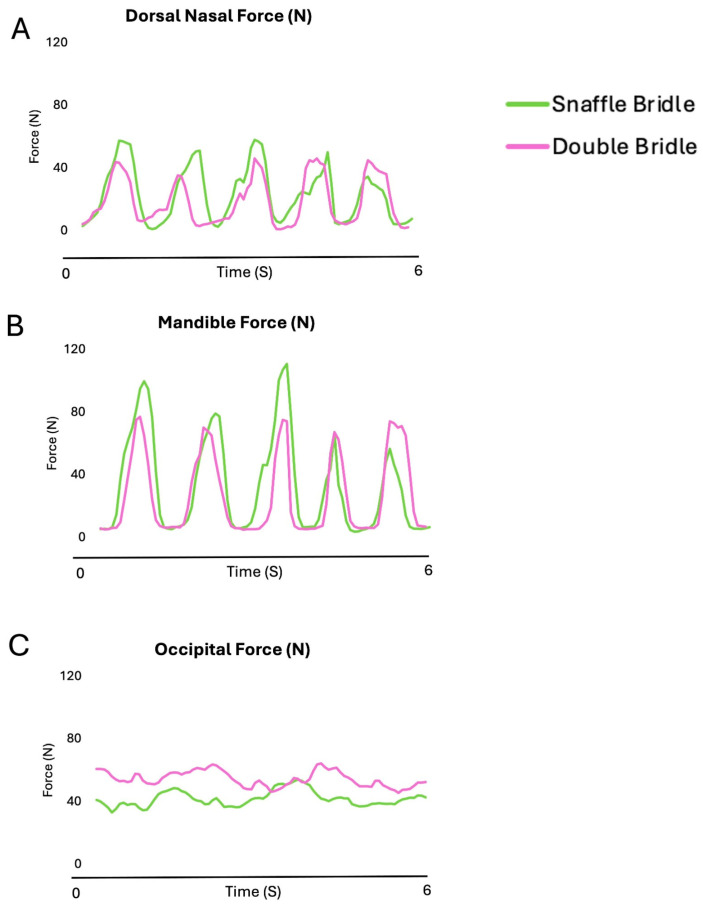
Bridle forces on the occipital region (**A**), nasal bones (**B**), and mandibular rami (**C**) for a single horse ridden in collected walk with a snaffle bridle (green line) and double bridle (purple line).

Figure 3 shows the oscillating pattern of the forces beneath the noseband and the headpiece of the SB and DB in five strides of collected walk. Increases in nasal and mandibular forces occur synchronously but with higher values on the mandibles. Occipital forces are usually highest, especially for the DB.

#### 3.1.2. Total Rein Tensile Forces (Tension)

**Figure 4:**
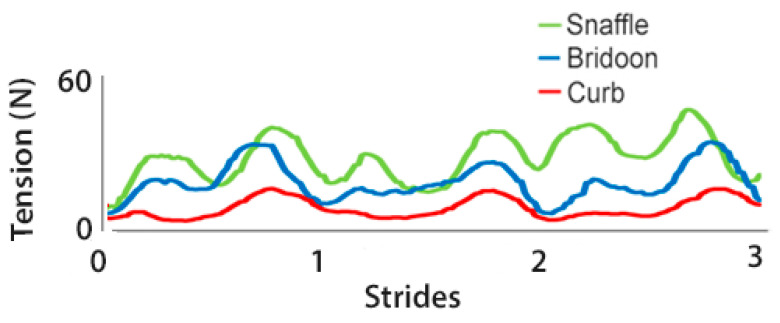
Rein tension summed over left and right reins for a single horse performing 3 strides of collected walk starting at right hind contact. Data show the snaffle bit of a snaffle bridle (green line) and the bridoon (blue line) and curb (red line) bits of a double bridle.

**Figure 5:**
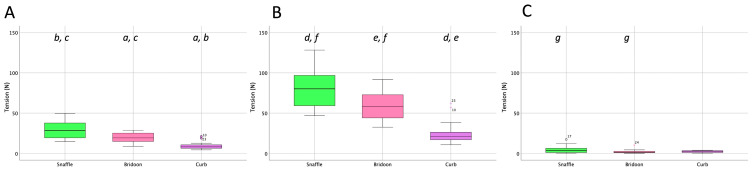
Box plots for collected walk illustrating (**A**) mean, (**B**) maximal, and (**C**) minimal total rein tension (sum of left and right rein forces) for the snaffle bit of a snaffle bridle (green) and the bridoon (pink) and curb (purple) bits of a double bridle. The central line is the median, the box represents the 25th and 75th percentiles, and the whiskers are the maxima and minima not considered outliers. ° represents outliers included in the analysis. Within each plot, boxes with the same letters differ significantly (*p* < 0.02).

The rein tension curve at walk showed two elevations per stride that occurred at the same time in the stride for the snaffle, bridoon, and curb reins. Through most of the stride, tension was highest in the snaffle rein and lowest in the curb rein (Figure 4). There were no significant differences between the SB and DB (bridoon and curb combined) in the mean, maximal, and total rein tension (Table 2 and Figure 5).

The total forces exerted by both reins of the snaffle, bridoon, and curb bits are shown in Figure 5. The mean, maximal, and minimal rein tension values were significantly higher for the snaffle than the bridoon. For the maximal and mean values only, tensions in the snaffle and bridoon reins were significantly higher than in the curb reins (*p* < 0.02) as indicated by the bold text. Superscripts indicate post hoc differences between bit types. 

#### 3.1.3. Bridle Pressure Distribution

**Figure 6:**
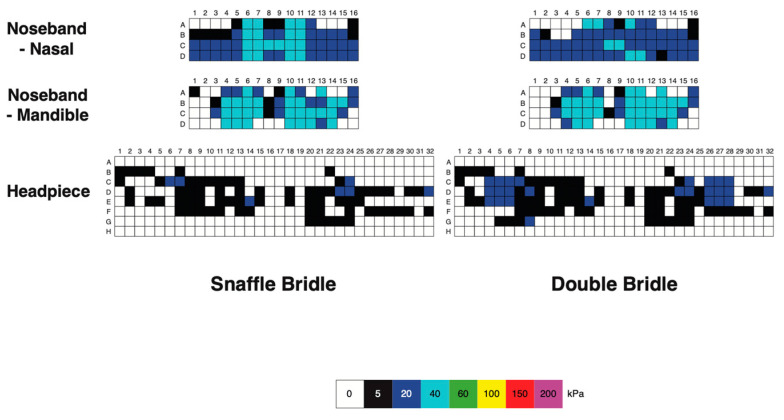
Pressure map of a single horse ridden at collected walk showing pressure on the nasal bones (upper row), mandibular rami (central row), and occipital region (lower row). Snaffle bridle scans are on the left and double bridle scans are on the right. Note the relatively unloaded areas of each scan on the midline.

Nasal, mandibular, and occipital pressure scans of each horse indicated that the force was not evenly distributed beneath the noseband or headpiece of the bridle but the pressure patterns were similar for the SB and DB (Figure 6). At the nasal location, pressure was typically concentrated over 2 cm wide areas on the lateral aspects of the nasal bones, where the pressure magnitude was 40–60 kPa. There was a less loaded area approximately 2 cm wide over the midline nasal suture. Each ramus of the mandible was loaded over an area about 3 cm wide, with pressure in the range of 60–200 kPa. The intermandibular space between the rami was less loaded (pressure ≤ 40 kPa) in this example. The occipital scans showed an unloaded area about 2 cm wide over the middle of the poll. Lateral to this, on each side was an area that was loaded fairly consistently at approximately 20 kPa, with a few sensors registering higher values up to 60 kPa (Figure 4).

**Figure 7:**
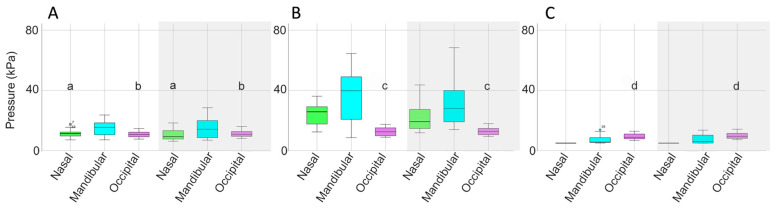
Box plots for collected walk illustrating (**A**) mean, (**B**) maximal, and (**C**) minimal pressures for the nasal (green), mandibular (cyan), and occipital (pink) sites. Each panel shows values for the snaffle bridle on the left and values for the double bridle on the right in the shaded area of the plot. The central line is the median, the box represents the 25th and 75th percentiles, and the whiskers are the maxima and minima not considered outliers. ° represents outliers included in the analysis. Similar superscripts for the snaffle bridle and the double bridle within the same graph indicate significant differences (*p* ≤ 0.02). Values shown in Table 3.

Mean, maximal, and minimal pressures at the nasal, mandibular, and occipital sites, with comparisons between the SB and DB are presented in Table 3 and Figure 7. Mean nasal pressure was higher (*p* = 0.02) when horses were ridden in the SB than the DB. Occipital mean, maximal, and minimal pressures were higher for DB than SB (*p* = 0.001, *p* = 0.010, *p* ≤ 0.001, respectively). Maximal mandibular pressures had particularly high variability (Table 3, Figure 7).

### 3.2. Collected Trot

#### 3.2.1. Bridle Forces

Comparisons at the nasal, mandibular, and occipital locations (Table 4) showed significantly higher mean, maximal, and minimal occipital forces for DB than SB (*p* < 0.001, *p* = 0.010, *p* = 0.009, respectively). There were no significant differences between bridles for the nasal and mandibular locations.

**Figure 8:**
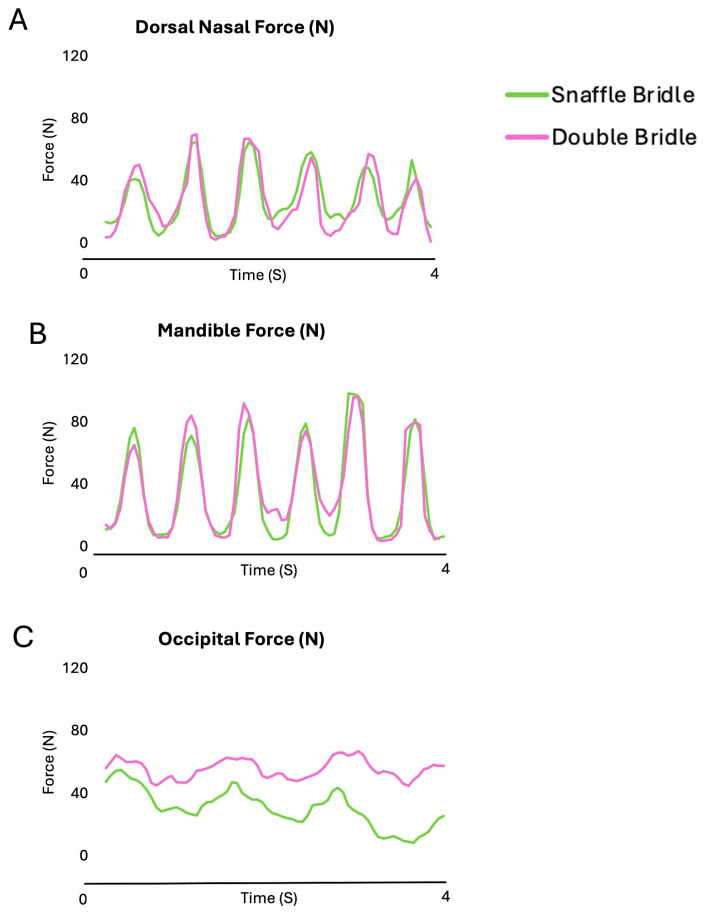
Bridle forces on the occipital region (**A**), nasal bones (**B**), and mandibular rami (**C**) for a single horse ridden in collected trot with a snaffle bridle (green line) and double bridle (pink line).

Figure 8 shows the force–time curves for the nasal and mandibular sites showed an undulating pattern, with two synchronous peaks per stride at the nasal and mandibular sites. Force at the occipital site increased early in diagonal stance.

#### 3.2.2. Total Rein Tensile Forces (Tension)

**Figure 9:**
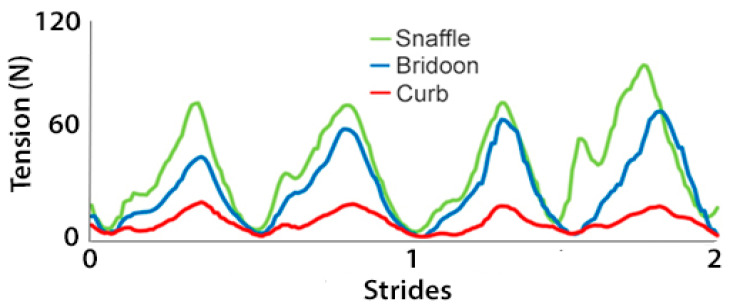
Rein tension data for a single horse during 2 strides of collected trot. Tensions are summed over left and right reins for the snaffle bit of a snaffle bridle (green line) and for the bridoon (blue line) and curb (red line) bits of a double bridle.

**Figure 10:**
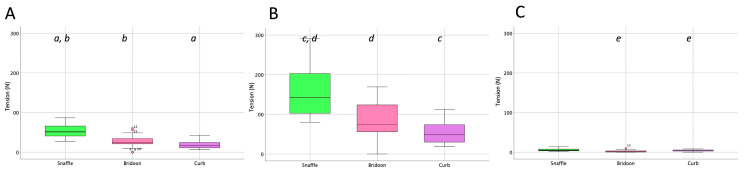
Box plots for collected trot (sitting) illustrating (**A**) mean, (**B**) maximal, and (**C**) minimal rein tension summed over the left and right reins for the snaffle (green), bridoon (pink), and curb (purple) bits. The central line is the median, the box represents the 25th and 75th percentiles, and the whiskers are the maxima and minima not considered outliers. ° represents outliers included in the analysis. Within each plot, boxes with the same letters differ significantly (*p* < 0.02).

Rein tension data at collected trot (Figure 9) showed two similar elevations per stride, one during each diagonal stance phase. The timing of the elevations was similar for the snaffle, bridoon, and curb reins, with the snaffle having the highest tension values and the curb having the lowest values. When rein tensions were compared between bridles, mean rein tension was significantly higher for the SB than the DB (Table 5 and Figure 10).

Tensile forces summed over the left and right reins for the three bits (Figure 10) demonstrated that the mean and maximal forces were significantly higher for the snaffle than for the bridoon or the curb (*p* < 0.02).

#### 3.2.3. Pressure Distribution

**Figure 11:**
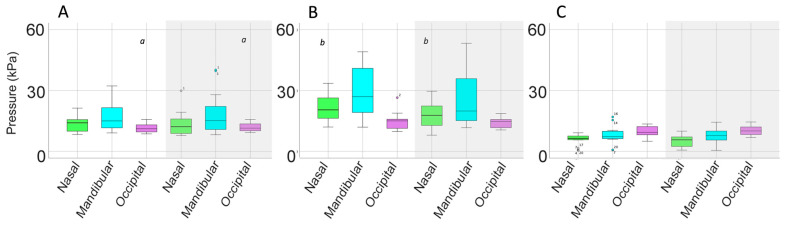
Box plots for collected trot illustrating (**A**) mean, (**B**) maximal, and (**C**) minimal pressures for the nasal (green), mandibular (cyan), and occipital (pink) sites. Values for the snaffle bridle are on the left and values for the double bridle are in the shaded area on the right of each plot. The central line is the median, the box represents the 25th and 75th percentiles, and the whiskers are the maxima and minima not considered outliers. ° represents outliers included in the analysis. Similar superscripts within the same graph indicate values that are significantly different between the two bridles (*p* ≤ 0.02). Values shown in Table 6.

**Figure 12:**
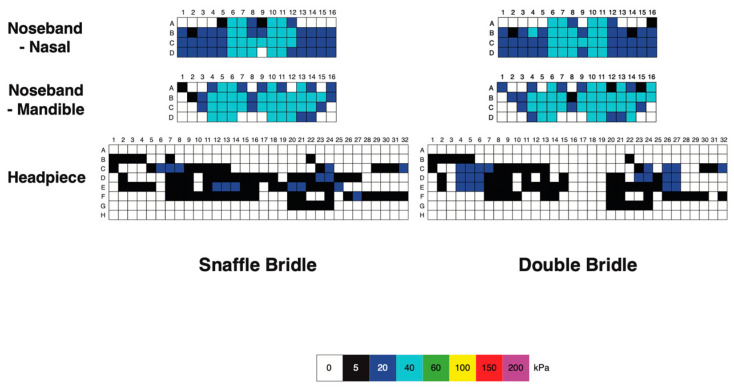
Pressure scans of a single horse ridden at collected trot showing pressure on the nasal bones (upper row), mandibular rami (central row), and occipital region (lower row). Snaffle bridle scans are on the left and double bridle scans are on the right for each site. Note the relatively unloaded areas of each scan on the midline.

Pressure distributions at the different sites indicated that mean occipital pressure was significantly higher for the DB (*p* = 0.003) and maximal nasal pressure was higher for the SB (*p* = 0.010) (Table 6, Figure 11 and Figure 12).

### 3.3. Collected Canter

#### 3.3.1. Bridle Forces

**Figure 13:**
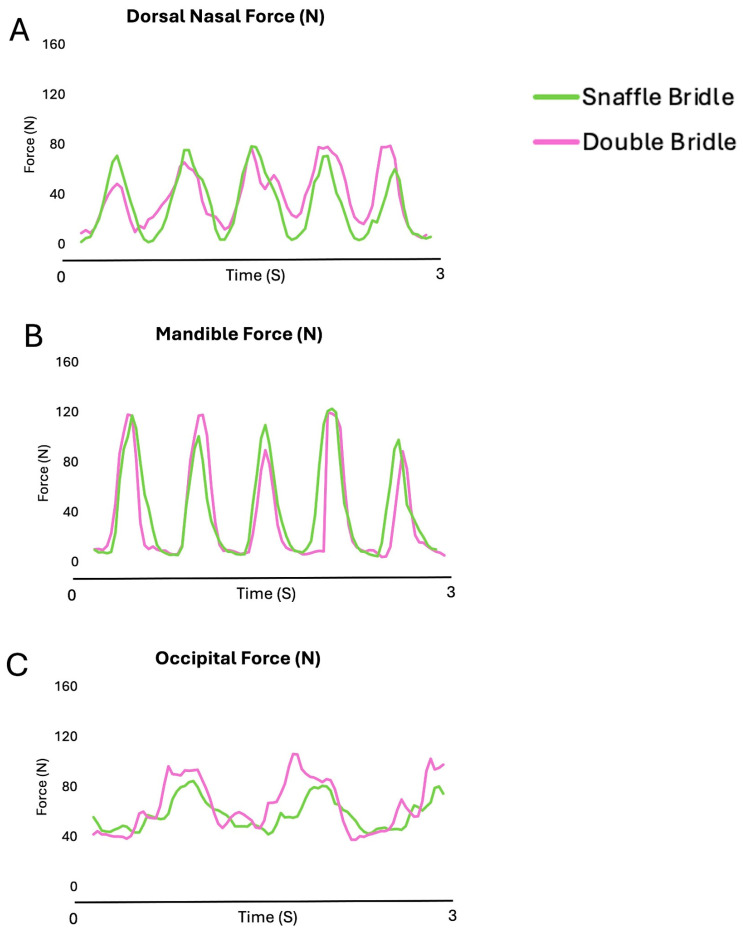
Bridle forces on the occipital region (**A**), nasal bones (**B**), and mandibular rami (**C**) for a single horse ridden in collected canter with a snaffle bridle (green line) and double bridle (pink line). Maximum forces occurred during diagonal stance in the occipital, with noseband forces occurring during the early stance phase of the trailing hind limb and during the second half of the leading forelimb stance phase.

During canter, forces on the nasal, mandibular, and occipital sites had an oscillating pattern (Figure 13). The maximal and mean occipital forces were significantly higher with the DB than the SB (*p* = 0.010, *p* < 0.001, respectively) (Table 7).

Figure 13 shows the force–time curves for the nasal and mandibular sites showed an undulating pattern. Maximum forces occurred during diagonal stance in the occipital, with noseband forces occurring during the early stance phase of the trailing hind limb and during the second half of the leading forelimb stance phase.

#### 3.3.2. Total Rein Tensile Force (Tension)

**Figure 14:**
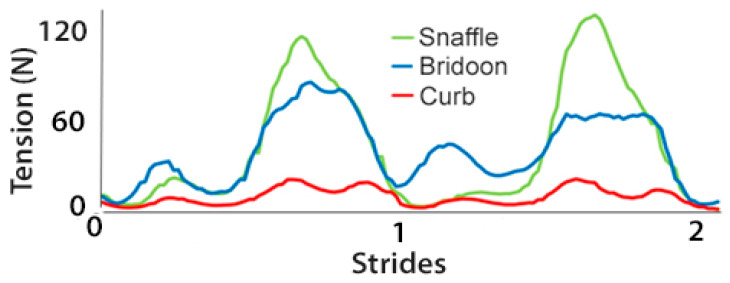
Rein tension for a single horse during 2 strides of collected canter. Tension values are summed over left and right reins for the snaffle bit of a snaffle bridle (green line) and for the bridoon (blue line) and curb (red line) bits of a double bridle.

**Figure 15:**
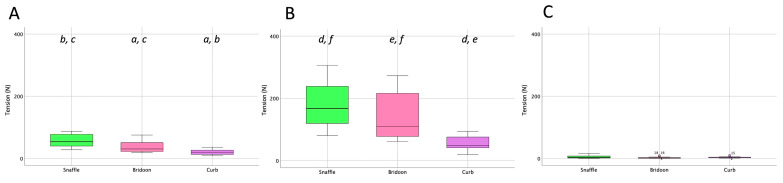
Box plots for collected canter illustrating (**A**) mean, (**B**) maximal, and (**C**) minimal total rein tension (left and right summed) for the snaffle, bridoon, and curb bits. The central line is the median, the box represents the 25th and 75th percentiles, and the whiskers are the maxima and minima not considered outliers. ° represents outliers included in the analysis. Within each plot, boxes with the same letters differ significantly (*p* < 0.02).

In collected canter, rein tension values in the snaffle and bridoon reins showed a small elevation during the trailing hind stance phase that tended to have the highest values in the bridoon rein. This was followed by a larger elevation that was highest in the snaffle rein. The curb rein maintained low tension, with three small elevations during the stance phases of the trailing hind, the diagonal limb pair, and the leading forelimb (Figure 14). The mean, maximal, and minimal total rein forces did not differ between the single bit of the SB and the summed values for the two bits of the DB (Table 8 and Figure 15).

#### 3.3.3. Pressure Distribution

**Figure 16:**
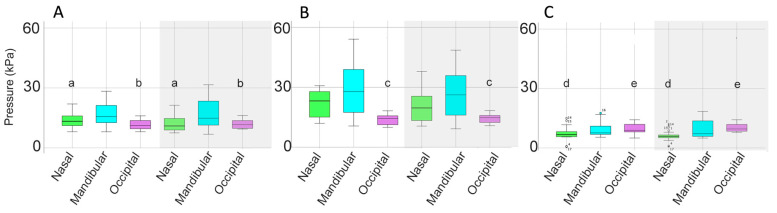
Box plots for collected canter illustrating (**A**) mean, (**B**) maximal, and (**C**) minimal pressures for the nasal (green), mandibular (cyan), and occipital (pink) pressure mats. In each panel, values for the snaffle bridle are on the left and values for the double bridle are on the right in the shaded area of the plot. The central line is the median, the box represents the 25th and 75th percentiles, and the whiskers are the maxima and minima not considered outliers. ° represents outliers included in the analysis. Similar superscripts within the same graph indicate values that are significantly different between the two bridles (*p* ≤ 0.02). Values are shown in Table 9.

**Figure 17:**
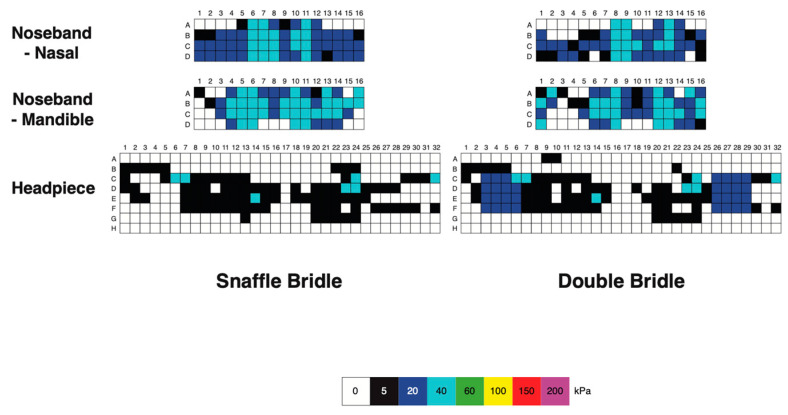
Pressure scans of a single horse ridden at collected canter showing pressure on the nasal bones (upper row), mandibular rami (central row), and occipital region (lower row). Snaffle bridle scans are on the left, and double bridle scans are on the right for each site.

The mean, maximal, and minimal bridle pressures at the nasal, mandibular, and occipital sites (Table 9, Figure 16 and Figure 17) show that mean and minimal nasal pressures were higher when horses were ridden in SB than DB (*p* = 0.002, *p* = 0.006, respectively). The mean, maximal, and minimal occipital pressures were higher for DB than SB (*p* < 0.001, *p* = 0.010, and *p* < 0.001, respectively).

## 4. Differences Between Gaits

For all force variables, walk had significantly lower values than trot and canter, which did not differ from each other. Mean pressure, except the SB at the occipital site, had significantly lower values for walk than trot or canter. The mean, maximal, and minimal pressures are shown in Table 10.

## 5. Discussion

This study compared forces and pressures associated with using an SB vs. a DB in high-level dressage horses and riders performing collected walk, trot, and canter. The quantitative data support our first hypothesis that occipital forces are higher for the DB than the SB. The second hypothesis, that sub-noseband pressures on the nasal and mandibular bones would be similar for SB and DB, was not supported. The third hypothesis, that rein tension values would be higher when ridden in a DB compared with an SB, was also not supported.

The headpiece transmits the weight of the bridle and bits to the occipital region of the horse. The DB was, on average, 0.86 kg heavier than the SB; this was associated with increases in the mean occipital force of 3.6 N in walk, 9.5 N in trot, and 5.0 N in canter. In order to dampen forces and reduce pressure on the occiput, it is recommended that the headpiece should be wide (relative to anatomy) to increase the area of force distribution and lined by a uniformly thick layer of resilient padding to absorb the force. Interestingly, pressure mapping across the occipital region indicated unequal loading across the occipital region, with a relatively unweighted area that extended about 3 cm either side of the midline. In a previous study [3], pressures during trotting were localised to the base of the ear which was loaded relatively consistently at about 20 kPa. We hypothesise that the inertial effect of the weight of the bits applies tension to the cheekpieces, which presses the base of the headpiece medially against the base of the ears. This, in turn, rounds the middle part of the arched headpiece, slightly elevating it and reducing pressure over the mid-occipital region and an unloaded area about 6 cm wide over the middle of the occiput, indicating that minimal pressure is applied in this area.

The SB and DB were fitted with identical Swedish nosebands adjusted to a 2.0 finger-equivalents tightness. Contrary to our second experimental hypothesis, different pressures were recorded for the two bridle types. The DB exerted lower mean nasal forces on the lateral part of the nasal bones in walk and canter. The DB also had lower mean forces over the mandibular rami, which has been shown previously to be more loaded than the nasal bones during standing and chewing a treat [32] and when riding in rising trot [19]. These findings indicate the need for effective padding over the mandibles.

In trot, the maximal occipital force (60.3 N) and pressure (14.7 kPa) measured in the present study are smaller than those reported in high-level dressage horses trotting with a double bridle (255.9 N, 46.5 kPa, respectively) [3]. Additionally, the maximal nasal force (18.4 N) and pressure (17.7 kPa) are considerably lower than the values of 64 N and 53 kPa, respectively, reported by Murray et al. [3]. Previously, we reported even lower nasal and mandible pressures for horses ridden in rising trot in an SB with a Swedish noseband adjusted to 2 finger-equivalents tightness [19]. Differences between pressure values may be related to the horse’s head position: nasal pressure increases with the head further in front of the vertical. Although head angles were not measured in this study, it might be expected that horses ridden in previous studies with less collection in an SB would have the head further in front of the vertical.

Determining the magnitude and duration of pressure at which horses feel discomfort is challenging. Pressure algometry is a repeatable and semi-objective technique to determine the mechanical nociceptive threshold (MNT) at which horses exhibit an avoidance response [33]. The MNT for the temporomandibular joint has been measured as being 500–600 kPa [34]. In the current study, across all gaits, the highest maximal noseband pressures were for the mandible in collected walk when wearing the SB (<40 kPa) and DB (<29 kPa). Whilst MNT values have not been reported for locations beneath the noseband, the values presented here are considerably lower than the MNT over the temporomandibular joint. The DB has bridoon and curb mouthpieces, which have been suggested to apply higher total force to the soft tissues of the mouth, including the tongue, mucosa overlying the mandibular diastema, and the lip commissures. Whilst the technology needed to measure intra-oral pressure is not available, we found no differences between the total force for the single bit of the SB compared with the two bits of the DB in the mean or maximal values of rein tension for horses ridden in collected walk and collected canter. In collected trot, the mean total force was actually higher for the SB than the DB, which is contrary to our third hypothesis. The median values for the mean combined left and right rein forces in an SB (walk: 28.5 N, trot: 48.2 N, canter: 53.7 N) are similar to the equivalent rein tension values for professionals riding in an SB with short reins at walk (24 N), sitting trot (36 N), and seated canter (44 N) [27].

The experience of the riders in this study is evident from Figure 4, Figure 9 and Figure 14 that show rein tension in walk, trot, and canter, respectively. The traces for the snaffle and bridoon show similar patterns but with lower overall tension for the bridoon reins in all gaits. Tension in the curb rein follows a similar pattern to the bridoon but has lower values and less obvious elevations. These findings indicate that the riders were able to control tension independently in the bridoon and curb reins, maintaining a light contact with the horse’s mouth as the rein tension values changed in accordance with the oscillations of the horses’ head and neck. It remains to be seen how less experienced riders perform under similar circumstances and whether they can differentiate equally well between tension applied to the bridoon and curb reins [29]. Furthermore, a horse with more advanced training may respond to lower and more subtle rein tension aids than a less trained horse, so it cannot be expected that the findings will necessarily be the same across the entire spectrum of training levels.

Mouthpieces vary in shape, width, and design, with straight bar curb bits being associated with oral lesions on the bars of the mandibles, whereas jointed bits are more often associated with lesions in the lip commissures [35]. The horses in our study were routinely ridden in both SB and DB several times per week and had been trained in a DB for at least 12 months. As part of the inclusion criteria, all horses had a dental assessment prior to the study, and no dental pathology or oral lesions were reported, which further attests to the riders’ skills. The bridles and bits in this study were adjusted by skilled professionals to ensure optimal fit, which is unlikely to be present in all horses.

In a multi-disciplinary study of horses and ponies competing in the FEI disciplines, the prevalence of oral lesions was highest in dressage horses/ponies and increased with competition level but did not differ between different types of bit or bitless bridles [36]. In the study reported here, no differences were found in mean or maximal rein tension between the combined curb and bridoon reins in the DB and tension in the snaffle rein of the SB. The relationship between the two bits of the DB within the horse’s oral cavity is complex. The bridoon is adjusted higher in the horse’s mouth and its mouthpiece lies on top of the curb mouthpiece [37], resulting in an interaction between the two mouthpieces [38]. Further study is needed to understand the interactions between the bit and the oral tissues, particularly pressures which are influenced by the contact area of the bit. For example, tightening of the curb chain affects rotation of the curb bit and the resulting magnitude and distribution of forces [38]. Additionally, the curb bit is not a fixed first-class lever which requires a fixed fulcrum. Instead, it has been described as having a floating fulcrum (allowing the lever to form and extend as needed [39]), which complicates the estimation of intra-oral pressure. Other factors that have an influence, including the horse’s head and neck position, bit design [40], contact area of the bit(s), tongue–bit interaction [38,41], and rein tension, are outside the scope of this study.

Limitations to the study include the fact that the riders’ requested that the noseband be adjusted to 2 finger-equivalents tightness throughout the study because this is the laxity the horses were accustomed to. It has been shown in a previous study that mean, maximal, and minimal nasal and mandibular pressures do not differ between noseband adjustments of 2.0 and 1.5 finger-equivalents tightness [19], but these findings may not apply to nosebands adjusted tighter than 1.5 finger-equivalents or looser than 2 finger-equivalents. Although the sample size was small, it provided 90% power. The subjects were a uniform population of horses and riders, who were all highly skilled and experienced, but caution should be used when applying these findings to less skilled horses and riders. While the results provide quantitative data that increase our understanding of the impact of SB and DB on horses, it should be noted that rein tension and pressure are elements of a complex and dynamic interaction between the horse, rider, and tack. At this time, measurements of intra-oral pressures in the horse are not reliable and accurate empirical data are required to fully understand the impact of different bits and bridles on the tissues of the horse’s mouth. The influence of the SB and DB on equine behaviour across the study is not reported here but is currently being analysed to consider the broader impact on the horse. Lastly, the horses’ own bridles and bits were used, meaning that the curb bit in particular was not standardized.

## 6. Conclusions

This study provides quantitative data from experienced dressage horses comparing forces on the head and rein tensions when ridden in an SB vs. a DB. Following the studied gaits, the findings revealed few differences between the SB and DB in pressures beneath the noseband but headpiece pressures were significantly higher for the DB due to the additional weight of the bridoon and curb bit. Occipital force and pressure can be ameliorated by minimising the weight of the bits and bridle and by using a wide headpiece padded with a uniformly thick layer of smooth, resilient padding. Interestingly, total rein force was similar for the SB and DB. The bridoon and curb reins had lower mean and maximal tension than the snaffle rein. These findings have not shown detrimental effects in terms of noseband pressures and rein forces associated with the use of a DB by appropriately skilled riders but it is not known whether less skilled horses and riders would perform equally well.

## Figures and Tables

**Table 1:** Median [25th and 75th] percentiles for the mean, maximal, and minimal total forces on the nasal, mandibular, and occipital sites for 11 horses ridden in collected walk. Probability values compare snaffle bridle and double bridle at each site. Bolded *p* values indicate significant differences (*p* ≤ 0.02) between bridle types.

Total Force	Location	Snaffle Bridle	Double Bridle	*p* Value
Mean (N)	Nasal	8.9 [4.1, 15.4]	5.6 [2.1, 18.8]	0.08
	Mandibular	14.8 [8.7, 31.5]	7.4 [4.0, 32.1]	0.03
	Occipital	32.2 [14.2, 39.1]	35.8 [23.9, 46.6]	**0.001**
Maximal (N)	Nasal	34.4 [8.2, 58.6]	13.7 [5.5, 46.1]	0.37
	Mandibular	55.1 [24.9, 110.8]	20.1 [13.4, 77.3]	0.21
	Occipital	33.9 [18.9, 55.3]	45.6 [32.1, 71.8]	**0.01**
Minimal (N)	Nasal	2.2 [1.2, 6.2]	2.2 [1.6, 3.2]	0.31
	Mandibular	6.3 [4.8, 16.1]	4.8 [1.2, 18.4]	0.37
	Occipital	22.8 [12.1, 42.5]	33.7 [19.4, 52.5]	**0.009**

**Table 2:** Median [25th and 75th] percentiles for mean, maximal, and minimal rein tension summed over left and right reins in 11 horses ridden at collected walk, superscripts indicate significant differences.

Rein Tension	SB Snaffle Bit	DB (Bridoon + Curb Bits)	*p* Value SB vs. DB	Curb Bit	Bridoon Bit	*p* Value (Curb vs. Bridoon vs. Snaffle)	Post Hoc
Mean (N)	28.5 [19.3, 38.8]	28.6 [22.6, 34.0]	0.67	8.1 [6.3, 11.0]	19.2 [14.8, 25.3]	**0.001**	**Curb < Bridoon *p* = 0.001 ^a^** **Curb < Snaffle *p* < 0.001 ^b^** **Bridoon < Snaffle *p* = 0.02 ^c^**
Maximum (N)	80.1 [57.4, 99.5]	78.9 [62.6, 109.6]	0.24	20.9 [16.8, 26.2]	58.5 [43.4, 73.4]	**<0.001**	**Curb < Snaffle *p* < 0.001 ^d^** **Curb < Bridoon P0.003 ^e^** **Bridoon < Snaffle *p* = 0.01 ^f^**
Minimum (N)	3.9 [1.1, 6.3]	3.7 [2.5, 6.1]	0.56	2.3 [1.3, 3.4]	1.5 [0.9, 2.6]	**0.01**	**Bridoon < Snaffle *p* = 0.01 ^g^**

**Table 3:** Median [25th and 75th] percentiles for mean, maximal, and minimal pressures at the nasal, mandibular, and occipital sites in horses ridden at collected walk with a snaffle bridle vs. a double bridle. Bold values and super scripts indicate significant differences (*p* ≤ 0.02) between bridle types.

Pressure	Location	Snaffle Bridle	Double Bridle	*p* Value
Mean (kPa)	Nasal	11.3 [9.3, 12.3]	9.1 [7.7, 13.5]	**0.02 ^a^**
	Mandibular	15.4 [10.3, 18.6]	13.5 [8.6, 20.0]	0.23
	Occipital	10.7 [9.0, 12.3]	11.0 [9.5, 12.7]	**0.001 ^b^**
Maximal (kPa)	Nasal	25.8 [17.5, 29.2]	19.4 [14.7, 27.9]	0.43
	Mandibular	39.7 [20.3, 49.1]	28.2 [18.9, 50.6]	0.98
	Occipital	12.5 [9.8, 15.1]	12.7 [10.5, 15.0]	**0.01 ^c^**
Minimal (kPa)	Nasal	5.0 [5.0, 5.0]	5.0 [5.0, 5.0]	1.0
	Mandibular	5.7 [5.5, 8.7]	6.0 [5.0, 10.3]	0.51
	Occipital	8.8 [7.7, 11.1]	9.4 [8.1, 11.5]	**<0.001 ^d^**

**Table 4:** Median values and [25th and 75th] percentiles for the mean, maximal, and minimal total forces on the nasal, mandibular, and occipital sites for horses ridden in collected trot. *p* values compare the snaffle bridle and double bridle at each site. Bolded *p* values indicate significant differences (*p* ≤ 0.02) between bridle types.

Total Force	Location	Snaffle Bridle	Double Bridle	*p* Value
**Mean** **(N)**	Nasal	15.8 [7.4, 29.3]	9.1 [5.2, 31.0]	0.27
	Mandibular	20.6 [9.1, 47.1]	20.2 [6.1, 46.2]	0.88
	Occipital	33.7 [17.7, 56.2]	43.2 [27.2, 61.4]	**<0.001**
**Maximal** **(N)**	Nasal	46.4 [19.3, 65.4]	18.4 [15.2, 74.9]	0.88
	Mandibular	85.1 [34.7, 98.0]	45.5 [28.2, 94.9]	0.76
	Occipital	47.3 [22.7, 77.3]	60.3 [39.0, 91.9]	**0.01**
**Minimal** **(N)**	Nasal	6.3 [1.6, 8.8]	5.0 [2.1, 9.2]	0.31
	Mandibular	8.1 [2.8, 24.0]	9.4 [2.6, 16.2]	0.67
	Occipital	28.5 [13.9, 50.6]	34.2 [19.6, 57.2]	**0.009**

**Table 5:** Median [25th and 75th] percentiles for mean, maximal, and minimal rein tensions summed over left and right reins in 11 horses ridden at collected trot. *p* values compare the snaffle bridle (snaffle bit) vs. double bridle (bridoon + curb bits). *p* values compare the two bridles and the three bits, with significantly different values in bold (*p* < 0.02), superscripts indicate significant differences.

	SB	DB	*p* Value	DB		*p* Value	
Rein tension	Snaffle	Bridoon + curb	SB vs. DB	Curb	Bridoon	(Curb vs. Bridoon vs. Snaffle)	Post Hoc
Mean (N)	48.2 [37.8, 60.2]	39.9 [33.4, 54.6]	**0.01**	14.2 [10.8, 20.7]	24.8 [20.4, 34.8]	**<0.001**	**Curb < snaffle** ***p* < 0.001 ^a^** **Bridoon < snaffle *p* < 0.001 ^b^**
Maximum (N)	142.4 [101.4, 204.3]	118.4 [96.3, 202.5]	0.20	48.5 [30.1, 75.8]	74.5 [55.0, 124.6]	**<0.001**	**Curb < snaffle** ***p* < 0.001 ^c^** **Bridoon < snaffle *p* < 0.001 ^d^**
Minimum (N)	4.6 [3.2, 7.8]	5.3 [3.4, 8.1]	0.69	3.3 [2.6, 5.4]	1.7 [0.3, 3.3]	**<0.001**	**Bridoon < curb** ***p* = 0.008 ^e^**

**Table 6:** Median [25th and 75th] percentiles for mean, maximum, and minimum pressures at the nasal, mandibular, and occipital sites in 11 horses ridden at collected trot with a snaffle bridle vs. a double bridle. Bold values indicate significant differences (*p* ≤ 0.02) between bridle types.

Pressure	Location	Snaffle Bridle	Double Bridle	*p* Value
Mean (kPa)	Nasal	14.0 [9.8, 15.6]	12.1 [8.8, 16.4]	0.10
	Mandibular	15.0 [11.5, 21.9]	15.1 [10.7, 22.2]	0.85
	Occipital	11.1 [9.5, 13.3]	11.4 [10.0, 13.6]	**0.003**
Maximal (kPa)	Nasal	20.4 [15.5, 26.6]	17.7 [12.7, 23.8]	**0.01**
	Mandibular	26.9 [18.6, 41.3]	19.8 [15.1, 36.0]	0.13
	Occipital	15.0 [11.1, 16.1]	14.7 [11.5, 16.0]	0.95
Minimal (kPa)	Nasal	6.4 [5.6, 7.5]	5.6 [1.7, 7.1]	0.17
	Mandibular	7.2 [6.1, 9.9]	7.7 [5.5, 10.3]	0.37
	Occipital	9.3 [8.1, 12.2]	10.1 [8.2, 12.1]	0.18

**Table 7:** Mean, maximal, and minimal total forces on the nasal, mandibular and occipital sites for horses ridden in collected canter. Bolded *p* values indicate significant differences (*p* ≤ 0.02) between the snaffle bridle and double bridle.

Total Force	Location	Snaffle Bridle	Double Bridle	*p* Value
Mean (N)	Nasal	12.7 [6.6, 30.2]	11.3 [3.1, 33.0]	0.88
	Mandibular	20.0 [10.2, 49.5]	30.3 [5.0, 49.8]	0.96
	Occipital	37.0 [20.9, 56.0]	42.0 [29.0, 71.8]	**<0.001**
Maximal (N)	Nasal	60.7 [21.6, 78.4]	75.3 [11.4, 78.1]	0.54
	Mandibular	76.2 [33.7, 125.5]	80.6 [24.3, 123.3]	0.76
	Occipital	58.3 [31.9, 83.9]	60.5 [46.1, 106.4]	**0.01**
Minimal (N)	Nasal	3.8 [3.1, 9.8]	2.5 [1.9, 11.4]	0.51
	Mandibular	12.1 [1.8, 22.4]	5.8 [2.4, 33.2]	0.21
	Occipital	29.4 [16.3, 44.1]	32.4 [20.3, 51.2]	0.04

**Table 8:** Median [25th and 75th] percentiles for minimum, maximum, and mean total rein forces (summed over left and right reins) in 11 horses ridden at collected canter. *p* values compare the snaffle bridle (snaffle bit) vs. double bridle (bridoon + curb bits). *p* values compare the two bridles and the three bits, with significantly different values in bold (*p* < 0.02), superscripts indicate significant differences.

	Snaffle Bridle	Double Bridle	*p* Value	Double Bridle		*p* Value	
Rein tension	Snaffle bit	Bridoon + curb bits		Curb bit	Bridoon bit	Curb vs. Bridoon vs. Snaffle	Post hoc
Mean (N)	53.7 [39.4, 77.5]	50.7 [36.0, 83.3]	0.62	19.4 [12.4, 26.7]	30.3 [22.0, 51.6]	**<0.001**	**Curb < bridoon, *p* = 0.02 ^a^** **Curb < snaffle, *p* < 0.001 ^b^** **Bridoon < snaffle, *p* = 0.005 ^c^**
Maximum (N)	167.1 [118.1, 240.5]	171.3 [118.9, 271.1]	0.06	48.6 [40.4, 78.1]	109.8 [77.1, 216.7]	**<0.001**	**Curb < snaffle, *p* < 0.001 ^d^** **Curb < bridoon, P0.002 ^e^** **Bridoon < snaffle, *p* = 0.008 ^f^**
Minimum (N)	3.6 [1.1, 8.3]	4.3 [2.7, 8.2]	0.26	2.4 [1.9, 4.1]	1.6 [0.4, 2.7]	**0.03**	

**Table 9:** Median [25th and 75th] percentiles for mean, maximum, and minimum pressures at the nasal, mandibular, and occipital sites in 11 horses ridden at collected canter with a snaffle bridle vs. a double bridle. Bold values indicate significant differences (*p* = ≤0.02) between bridle types.

Pressure	Location	Snaffle Bridle	Double Bridle	*p* Value
Mean (kPa)	Nasal	13.2 [11.0, 16.4]	10.9 [8.8, 14.8]	**0.002**
	Mandibular	15.6 [12.5, 21.6]	14.8 [11.0, 23.4]	0.21
	Occipital	11.1 [9.5, 13.6]	11.5 [9.7, 13.6]	**<0.001**
Maximal (kPa)	Nasal	23.1 [14.9, 27.8]	19.6 [12.9, 26.4]	0.10
	Mandibular	27.8 [17.1, 40.0]	26.2 [15.5, 36.4]	0.31
	Occipital	14.4 [11.2, 15.6]	14.8 [11.4, 15.9]	**0.01**
Minimal (kPa)	Nasal	6.7 [5.6, 8.3]	5.8 [5.0, 6.8]	**0.006**
	Mandibular	7.5 [6.8, 11.9]	7.2 [5.7, 13.7]	0.35
	Occipital	8.9 [77.9, 11.9]	9.5 [8.4, 11.8]	**<0.001**

**Table 10:** Differences in force and pressure variables between gaits at the nasal, mandibular, and occipital sites in horses ridden with a snaffle bridle vs. a double bridle (N = 11). NS = non-significant values *p* > 0.02, superscripts indicate significant differences.

Variable	Bridle	*p* Value	Post Hoc
Nasal force (N)	Snaffle	<0.001	Walk < canter, *p* = 0.005 Walk < trot, *p* = 0.001
	Double	<0.001	Walk < canter, *p* = 0.003 Walk < trot, *p* = 0.002
Mandibular force (N)	Snaffle	0.001	Walk < canter, *p* = 0.008 Walk < trot, *p* = 0.003
	Double	<0.001	Walk < canter, *p* = 0.003 Walk < trot, *p* = 0.002
Occipital force (N)	Snaffle	<0.001	Walk < canter, *p* = 0.001 Walk < trot, *p* < 0.001
	Double	<0.001	Walk < canter, *p* < 0.001 Walk < trot, *p* < 0.001
Nasal mean pressure (kPa)	Snaffle	<0.001	Walk < canter, *p* = 0.002 Walk < trot, *p* = 0.003
	Double	0.002	Walk < canter, *p* = 0.02 Walk < trot, *p* = 0.002
Mandibular mean pressure (kPa)	Snaffle	0.003	Walk < canter, *p* = 0.01 Walk < trot, *p* = 0.008
	Double	0.003	Walk < canter, *p* = 0.02 Walk < trot, *p* = 0.005
Occipital mean pressure (kPa)	Snaffle	0.17	-
	Double	<0.001	Walk < canter, *p* < 0.001 Walk < trot, *p* = 0.005
Nasal maximal pressure (kPa)	Snaffle	0.71	-NS
	Double	0.24	-NS
Mandibular maximal pressure (kPa)	Snaffle	0.27	-NS
	Double	0.006	Walk > trot, *p* = 0.01 Walk < canter, *p* = 0.01
Occipital maximal pressure (kPa)	Snaffle	0.010	Walk < canter, *p* = 0.01
	Double	<0.001	Walk < canter, *p* = <0.001 Walk < trot, *p* = 0.006
Nasal minimal pressure (kPa)	Snaffle	<0.001	Walk < canter, *p* = 0.01 Walk < trot, *p* = 0.002
	Double	0.020	Walk < canter, *p* = 0.02
Mandibular minimal pressure (kPa)	Snaffle	<0.001	Walk < canter, *p* < 0.001 Trot < canter, *p* = 0.001^t^
	Double	<0.001	Walk < canter, *p* = 0.02^u^ Trot < canter, *p* < 0.001^v^
Occipital minimal pressure (kPa)	Snaffle	0.15	-NS
	Double	0.10	-NS

## Data Availability

Data are unavailable due to privacy or ethical restrictions.
